# SELDI-TOF analysis of glioblastoma cyst fluid is an approach for assessing cellular protein expression

**DOI:** 10.1179/016164113X13756993777580

**Published:** 2013-12

**Authors:** Martin Hoelscher, Nina Richter, Christian Melle, Ferdinand von Eggeling, Anne Schaenzer, Ulf Nestler

**Affiliations:** 1 Department of Neurosurgery, Justus Liebig University, Giessen, Germany; 2 Institute of Human Genetics, CUCA, Jena University Hospital, Jena, Germany; 3 Institute for Neuropathology, Justus Liebig University, Giessen, Germany

**Keywords:** Basigin, Cystic glioblastoma, Ferritin, Metallothionein, SELDI-TOF

## Abstract

**Objectives:**

In about 10% of glioblastoma patients, preoperative MRI discloses the presence of tumor cysts. Whereas the impact of cystic appearance on prognosis has been discussed extensively, only little is known about the tumor cyst fluid. In this study, we tested the feasibility of the surface enhanced laser desorption ionization time of flight (SELDI-TOF) technique to detect cyst fluid proteins.

**Methods:**

Cyst fluid was collected from 21 glioblastoma patients for SELDI-TOF analysis and compared to control cerebrospinal fluids from 15 patients with spinal stenosis. Resulting protein peaks with significant differences between groups were further described, using the molecular weight in an internet search of protein databases and publications. Two potential cyst fluid proteins, basigin and ferritin light chain, were selected for immunohistological detection in the histologic slides of the patients, metallothionein (MT) served as negative control.

**Results:**

As supposed from the results of the SELDI-TOF analysis, basigin and ferritin were detected immunohistochemically in the cyst wall, whereas MT was more equally distributed between the cyst wall and the surrounding tumor tissue. Median survival time of the patients was 20 months (range 2 to 102 months) and correlated with age, but not with expression of the three proteins.

**Discussion:**

The SELDI-TOF approach reveals a number of proteins, potentially present in glioblastoma cyst fluid. Identification of these proteins in tumor cells may help understand the pathogenetic pathways and the prognostic value of cystic changes.

## Introduction

In patients with histological diagnosis of glioma WHO IV°, the appearance of tumor cysts on preoperative MRI is not rare. According to the literature, cystic tumors are encountered in 7% to 13% of glioblastoma cases. Cystic features have also been observed in a number of entities, such as meningioma, hemangioblastoma, or metastatic tumors.^1^ While cystic low-grade gliomas have been associated with increased postoperative survival compared to non-cystic low-grade gliomas, this is a matter of debate for cystic glioblastoma compared to non-cystic glioblastoma.2,^3^

Histologic evaluation of the cyst wall helps to distinguish between ‘real’ cysts with an endothelial coating and ‘pseudocysts’ with palisading cellular structures around the cavity.^4^ The cavity itself cannot be assessed histologically, because of loss of the cyst content during fixation and preparation. Cyst fluid remains difficult to obtain and to examine, because most samples are small and the protein concentration is not sufficient for standard examinations such as western blot.

This is why, only little information about the cyst fluid is available and the hypotheses for cyst formation include necrobiotic degradation of tumor tissue, active secretion of proliferative factors by the tumor cells, or mere trapping of cerebrospinal fluid (CSF).^5^^–^7 Depending on the way of cyst formation, the content of the cyst fluid is supposed to include apoptotic pathway proteins, e.g. TNF, Fas, or caspases, high concentrations of specific secreted proteins such as VEGF or TGF-beta, or a protein content very similar to that of CSF.

It has been shown that in the case of active secretion of proteins into the cystic cavity, the identification of the cyst content can improve knowledge on pathophysiologic pathways of the surrounding glioblastoma cells, which results in valuable hints for treatment strategies.^7^

In this study, in order to examine the protein content in detail, we introduce the surface enhanced laser desorption ionization time of flight (SELDI-TOF) analysis of glioblastoma cyst fluid. The technique needs only small amounts of sample, allows for protein-screening, and delivers protein weights with a high exactitude.^8^ The results are compared to the SELDI-TOF analysis of CSF specimens from tumor-free patients.

The SELDI-TOF mass spectroscopy is a discovery oriented approach that delivers a huge number of detectable protein sizes. When studying a single spectroscopic size, there are several proteins that could have been at the origin of the peak. In order to determine which of the corresponding proteins are present in the cyst fluid, immunohistological staining of the surrounding cyst wall has to be performed. Two potential candidates, basigin and ferritin, and one negative control, metallothionein (MT), were examined histologically in this study.^9^^–^11

## Methods

### Surface enhanced laser desorption ionization time of flight

#### Sample collection

Upon planning the neurosurgical resection or biopsy, informed consent from patients with cystic brain tumors was obtained to withdraw and store tumor cyst fluid for research purposes. During the intervention the cyst was punctured under ultrasonographic control or stereotactically, and the fluid was stored at 4°C for a few hours before being centrifuged. The supernatant was frozen at −20°C until use.

Altogether, cyst fluids from 18 glioblastoma patients and 7 recurrent glioblastoma were analyzed using SELDI-TOF. From these, 16 glioblastoma specimens and 5 recurrent glioblastoma were available for histologic immunostaining. Among these 21 cases 11 were male and 10 were female patients, mean age was 53·9 years (range 21 to 77) at the time of first operation.

As a control, 15 patients undergoing myelography because of spinal stenosis gave their informed consent for CSF withdrawal. The specimens were prepared as above.

#### Processing

**Figure 1:**
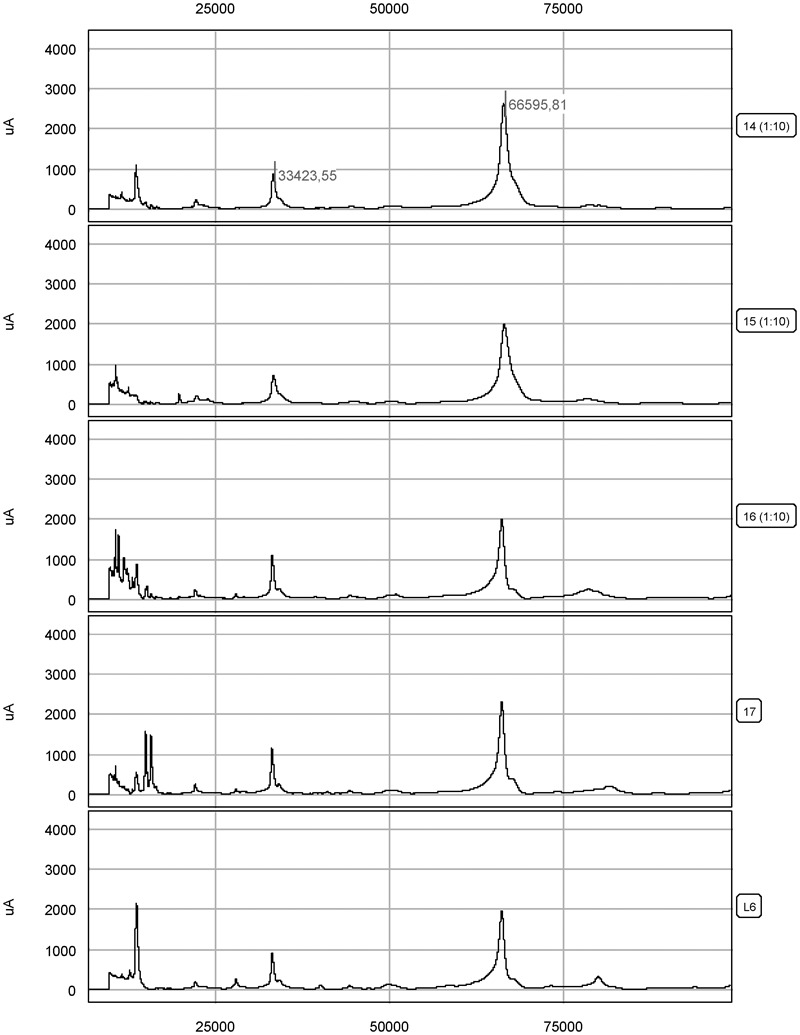
SELDI-TOF chromatographic results for four tumor cyst fluids #14 to #17 and one cerebrospinal fluid control #L6. The molecular weight of the peaks in daltons is given on the horizontal axis and the height of the peaks can be compared in micro-amperes on the vertical axis, as shown here for albumin and double loaded albumin.

Directly before use, the specimens were thawed and diluted 1:10 in buffer solution. Two microliters of the solution were pipetted on the chromatographic matrix slot and given time to be absorbed. The SELDI-TOF mass spectroscopy (ProteinChip technology) uses the affinity chromatographic surfaces to bind proteins specifically.^8^ Desorption in an electric field using a laser beam enables a mass spectrometric analysis that results in a protein spectrum with calculation of molecular weights with an accuracy of 0·3% (Fig. 1).

#### Statistical work-up

After determination of the different protein peaks in all samples, a computer-based statistical analysis of peak intensities was performed. At first, spectra were normalized towards each other and checked for data quality and plausibility. Then molecular masses and intensities of the peaks were compared between non-tumor CSFs and different tumor cyst fluids, grouped according to the underlying histological diagnosis. Protein sizes were listed when present in tumor cyst fluid and absent in control CSFs or *vice versa* (Table 1). The probability for a coincidental difference was calculated, taking into account the number of analyzable specimens per group and the molecular weight of each protein.^8^ Protein sizes were retained when a *p*-value below 0·05 was found.

**Table 1:** Comparing the presence of proteins in glioblastoma cyst fluid with control CSF, a significant difference was found for 51 peaks. Twenty-eight proteins were present in glioblastoma but absent in CSF. Glial fibre acid protein (GFAP) (49 880 Da) lies in the 0·3% range of the 49 916 peak and VEGF121 (17 219 Da) in the range of the 17 224 peak. The molecular mass of 19 899 Da corresponds to the ferritin light chain. The protein basigin was detected at 40 211 Da in recurrent glioblastoma when compared to glioblastoma cyst fluid (data not shown)

Glioblastoma *versus* CSF			CSF *versus* glioblastoma		
Signal found in	MW (Da)	*p*-value	Signal found in	MW (Da)	*p*-value
			CSF	3892	4·31×10^−4^
gbm cyst fluid	6424	4·42×10^−7^			
gbm cyst fluid	6633	1·53×10^−5^			
			CSF	6799	1·08×10^−3^
			CSF	6846	6·15×10^−3^
			CSF	6888	1·47×10^−4^
			CSF	6911	1·32×10^−5^
			CSF	6936	3·82×10^−6^
			CSF	7647	5·24×10^−6^
gbm cyst fluid	7921	3·46×10^−2^			
gbm cyst fluid	8633	4·48×10^−6^			
gbm cyst fluid	8687	3·82×10^−6^			
gbm cyst fluid	8899	9·83×10^−5^			
gbm cyst fluid	9362	3·26×10^−6^			
gbm cyst fluid	9418	3·26×10^−6^			
gbm cyst fluid	9948	4·42×10^−7^			
gbm cyst fluid	10 829	5·56×10^−3^			
			CSF	11 571	2·48×10^−4^
			CSF	11 679	2·36×10^−6^
gbm cyst fluid	12 559	2·77×10^−6^			
			CSF	13 687	3·69×10^−5^
			CSF	13 737	2·18×10^−4^
			CSF	13 775	5·65×10^−5^
			CSF	13 821	1·78×10^−5^
			CSF	13 879	9·74×10^−6^
			CSF	14 006	6·75×10^−4^
gbm cyst fluid	14 946	4·08×10^−3^			
gbm cyst fluid	15 118	1·00×10^−2^			
gbm cyst fluid	15 842	9·12×10^−3^			
gbm cyst fluid	16 656	1·33×10^−2^			
gbm cyst fluid	17 224	6·24×10^−7^			
gbm cyst fluid	17 372	1·70×10^−6^			
gbm cyst fluid	19 899	1·32×10^−5^			
gbm cyst fluid	21 615	2·07×10^−2^			
			CSF	22 884	8·37×10^−5^
			CSF	26 687	1·06×10^−2^
gbm cyst fluid	27 904	3·35×10^−3^			
gbm cyst fluid	28 056	1·61×10^−5^			
gbm cyst fluid	28 256	7·27×10^−6^			
gbm cyst fluid	28 918	2·98×10^−5^			
			CSF	34 268	3·79×10^−4^
			CSF	40 051	1·17×10^−5^
gbm cyst fluid	43 354	2·70×10^−6^			
gbm cyst fluid	47 111	2·68×10^−3^			
gbm cyst fluid	49 916	1·29×10^−2^			
			CSF	67 937	4·18×10^−3^
			CSF	73 115	9·66×10^−5^
			CSF	80 046	1·88×10^−5^
gbm cyst fluid	94 289	6·35×10^−4^			
gbm cyst fluid	122 567	6·41×10^−3^			
			CSF	146 376	2·68×10^−3^

CSF, cerebrospinal fluid; gbm, glioblastoma; MW (Da), molecular weight in daltons; *p*-value, level of significance in exponential form.

In this way a list of protein peaks, classified by their molecular weight and stratified by *p*-values, was obtained (Table 1). To identify which proteins were probably apt to have resulted in the spectroscopic peaks, an internet based search in the SwissProt database using the TagIdent tool was performed. The search was restricted concerning the species (*Homo sapiens*) and the isoelectric point (7·45, but allowing for a broad variation of ±5·0). Finally, the molecular weight of the spectroscopic peak was entered, accepting potential proteins in the range of ±0·3% of the mass, according to the accuracy of the SELDI-TOF measurements.

The TagIdent tool revealed several potential candidate proteins per peak. In order to further restrict this list of potential candidates, a Pubmed search was added. If publications concerning the protein were found, one point each was attributed for the following items: (i) protein is secreted or is a membrane protein, (ii) protein has been described in (healthy) brain, (iii) protein has been described in tumor tissue, and (iv) protein has been described in brain tumor tissue. By retaining the proteins which reached the most points, the algorithm reduced the potential candidate proteins to less than five in most peaks (Table 2). With this algorithm we postulated the presence of basigin and ferritin in the cyst wall, whereas MT did not fulfill the criteria.

**Table 2:** The 10 protein peaks present in glioblastoma and absent in CSF with the highest significance values, together with some of their candidate proteins. TagIdent tool identification used the molecular weight and a Pubmed search examined the relationship of the candidate to brain, to tumor, and to brain tumor

	Glioblastoma *versus* CSF							
	*p*-value	Mw [Da]	Mw candidate	Name of candidate protein	pI	Amino acids	Chromosome	Characteristics
1	0·442×10^−6^	6424	6437	Leucine zipper protein 6	9·69	58	7q33	Associated with myeloproliferative disease, highly expressed in brain tissue
								
2	0·442×10^−6^	9948	9933	Isoform 6 of laforin	9·05	88	6q24	Defects cause Lafora epilepsy
			9942	Cocaine- and amphetamine-regulated transcript protein	7·75	89	5q13·2	Hypothalamic satiety factor
			9967	Isoform 3 of coxsackievirus and adenovirus receptor	5·23	89	21q21·1	Secreted cell adhesion molecule
								
3	0·624×10^−6^	17 224	17 196	Tumor necrosis factor ligand superfamily member 12, secreted form	9·61	156	17p13·1	Promotes endothelial cell proliferation, highly expressed in brain tissue
			17 219	Isoform VEGF121 of vascular endothelial growth factor A	6·49	147	6p12	Acidic growth factor, freely secreted
			17 231	Contingent replication of cDNA 4	6·4	156	1q22	Promotes FOS promoter transcription, expressed in brain tissue
								
4	1·70×10^−6^	17 372	17 329/17 401	Isoform 4 and 5 of transforming growth factor alpha	7·9	162/163	2p13	Promotes cell proliferation, binds to EGF receptor
			17 353	Tumor necrosis factor, soluble form	6·99	157	6p21·3	Secreted part of proteolytically cleaved TNF-alpha
			17 376	Activation peptide fragment 1 of prothrombin	5·01	155	11p11	Secreted, involved in hemostasis
			17 377	Interleukin-1 beta	5·91	153	2q14	Secreted, probably released by damaged cells
			17 387	Interleukin-7	8·71	152	8q12–q13	Secreted, hematopoietic growth factor
								
5	2·70×10^−6^	43 354	43 349	Haptoglobin	6·13	388	16q22·1	Secreted tetramer, binds hemoglobin
			43 369	P2X purinoceptor 4	8·28	388	12q24·32	Receptor for ATP
								
6	2·77×10^−6^	12 559	12 553	Thy-1 membrane glycoprotein	9·17	111	11q22·3–q23	Antigen CD90, synaptogenesis in brain
			12 595	Dipeptidyl-peptidase 1 exclusion domain chain	4·95	110	11q14·2	Cathepsin C
								
7	3·26×10^−6^	9362	9370	NADH dehydrogenase 1 alpha subcomplex subunit 4	9·41	81	7p21·3	Mitochondrial respiratory chain, ubiquinone
			9377	Isoform 8 of forkhead box protein P2	5·64	87	7q31·1	Transcriptional repressor with strong expression in parts of the developing brain
			9390	Isoform 3 of tumor necrosis factor receptor superfamily member 6	9·19	86	10q24·1	Secreted, blocks apoptosis
								
8	3·26×10^−6^	9418	9419	C–X–C motif chemokine 14	9·9	77	5q31·1	Secreted, chemoattractant for neutrophils
			9425	Parathyroid hormone	9·1	84	11p15·2	Secreted hormone
								
9	3·82×10^−6^	8687	8671	Isoform 2 of high mobility group protein 20A	3·88	81	15q24	Plays a role in neuronal differentiation
			8685	C–C motif chemokine 2	9·39	76	17q11·2–q12	Secreted, chemoattractant for monocytes and basophils
			8708	Apolipoprotein A-II	5·05	77	1q21–23	Secreted, stabilizes HDL structure
								
10	4·48×10^−6^	8633	8614	Protein BRICK1	5·36	74	3p25·3	Required for cell proliferation
			8642	NADH dehydrogenase 1 beta subcomplex subunit 2	4·4	72	7q34–q35	Mitochondrial respiratory chain, ubiquinone
			8646	C–X–C motif chemokine 10	10·19	77	4q21·1	Secreted, chemoattractant for monocytes and T-lymphocytes
			8656	Isoform H of Ras association domain-containing protein 1	4·2	75	3p21·2	Potential tumor suppressor
			8658	Calcium/calmodulin-dependent protein kinase II inhibitor 2	5·31	79	3q27·1	Translates intracellular calcium changes associated with glioma cell migration

#### Immunohistology

In order to verify this approach to glioblastoma protein expression, we analyzed the presence of candidate proteins basigin and ferritin immunohistochemically in paraffin embedded slides for the 21 cystic glioblastoma patients. As control, MT was stained. Monoclonal mouse antibodies against metallothionein MT-1 and MT-2 (ab12228) and basigin (ab49493), as well as a rabbit-polyclonal antibody against ferritin (ab76768) were purchased from abcam (Cambridge, UK).

Basigin staining: the primary antibody is a monoclonal mouse antibody recognizing the extracellular domain of the protein. After 15 minutes of heat antigen unmasking in 0·01 mol/l citric acid monohydrate buffer (pH 6·0, Sigma, Germany), PBS washing and peroxidase blocking for 7 minutes in 3% H_2_O_2_ were performed. After washing with aqua destillata and PBS, incubation with normal goat serum 1:20 followed for 20 minutes. The primary antibody (1:50) was incubated for 1 hour at room temperature, followed by washing in PBS and visualization with the streptavidin-biotin HRP-duet system (DAKO, Denmark), which detects primary antibodies from mouse or rabbit.

Ferritin staining: the primary polyclonal rabbit antibody is directed against purified mitochondrial ferritin, which is a 24-oligomer of ferritin heavy chains and light chains in varying numbers. Heat antigen unmasking for 15 minutes in 0·001 mol/l EDTA buffer (pH 8·0, Sigma), PBS washing, peroxidase blocking, and normal goat serum blocking were performed. The primary antibody (1:500) was incubated for 1 hour at room temperature, the HRP-duet system was used for staining.

Metallothionein staining: the primary mouse monoclonal antibody reacts with MT-1 and MT-2. Without heat unmasking, the peroxidase blocking and normal goat serum were applied as above. The primary antibody (1:100) was incubated for 1 hour at room temperature, HRP-duet staining followed.

**Figure 2:**
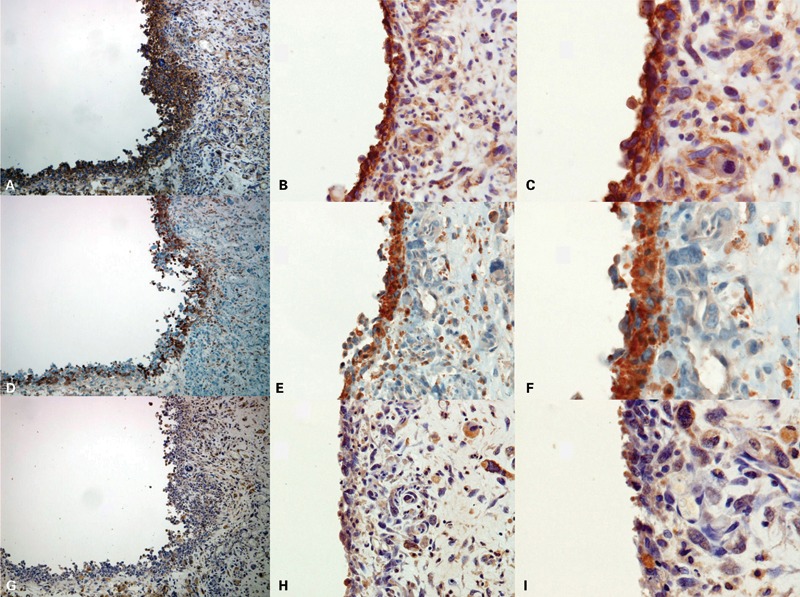
A–C) Basigin (CD147) staining of the cyst wall (original magnification 10×, 20×, and 40×, respectively). (D–F) Ferritin staining. (G–I) MT staining. Immunohistologic staining of two different glioblastoma cyst specimens (case 1: A, D, G and case 2: B, C, E, F, H, I). Basigin and ferritin are strongly expressed in the cyst walls, whereas metallothionein (MT) staining is less prominent and more equally distributed between the cyst wall and the main tumor tissue.

A counterstain with hemalaun was added (Fig. 2). Histologic slides of pulmonary carcinoma, human liver, and astrocytoma tissue served as positive controls for basigin, ferritin, and MT, respectively. The same specimens were stained without application of the first antibodies as negative controls.

All tumors were diagnosed in accordance with the WHO classification by a neuropathologist. One hundred and sixty-five tumor cysts were examined in the paraffin embedded specimens of the 21 patients, with a mean value of 4·2 cysts per patient. The immunostained sections were reviewed by one examiner (HM) after developing a standardized protocol with a semiquantitative scale from 0 to +++ for staining intensities. After the intensity of the cyst wall had been scaled, surrounding tumor tissue was examined using 20 fields of view and these staining intensities were averaged. The quotient, obtained by dividing the value of staining intensity in the cyst wall by the averaged value in surrounding cells, was calculated for each of the three proteins. In this way, predominance of the protein in the cyst wall was depicted by a quotient>1. For patients with multiple cysts in the paraffin slices, the quotients for each cyst were determined and then a mean cyst quotient was calculated for the patient.

To assess a probable impact of protein expression or age on survival since first surgery, statistical analysis was performed using the unpaired t-test. Patients were subgrouped into short-term survivors (up to 6 months) and long-term survivors (more than 30 months), and their results were compared to those of the opposite group, as well as to those of the cohort (Table 3).

**Table 3:** Staining quotients in 165 cysts. Mean staining quotients and mean age in patients, grouped according to survival time since first operation

Staining quotient: cyst wall versus tumor tissue in 165 cysts					
		Basigin	Ferritin	Metallothionein	
Cysts with quotient > 1		139 (84%)	107 (65%)	77 (47%)	

Mean staining quotient in patient tissues					
	Number	Basigin	Ferritin	Metallothionein	Mean age (years)
All patients	21	1·48	1·30	1·09	53·9
					
Surviving 1–6 months	6	1·39	1·36	1·267	69·3
Surviving 7–12 months	1	1·42	1·20	0·91	44·0
Surviving 13–18 months	3	1·47	1·13	0·96	67·0
Surviving 19–24 months	5	1·43	1·42	1·14	44·0
Surviving 25–30 months	2	1·40	1·68	1·08	31·0
Surviving>30 months	4	1·53	1·27	1·09	47·3

## Results

The SELDI-TOF analysis of the glioblastoma specimens resulted in a list of 51 protein peaks with significant difference between glioblastoma cyst fluid and control CSF. Among them, 28 proteins were present in glioblastoma cyst fluid and 23 in CSF (Table 1).

In a similar way, cyst fluid from recurrent glioblastoma was compared to CSF and to cyst fluid from glioblastoma (data not shown). In this dataset the protein peak at 40 211 Da is supposedly basigin. It was the only peak to be significantly more often present in recurrent glioblastoma fluid than in glioblastoma cyst fluid (*p * =  0·025).

Protein database and literature searches allowed identification of proteins potentially present in cyst fluid (Table 2). Several peaks could be directly attributed to proteins which have already been described in glioblastoma cyst fluid. This is the case for GFAP (peak at 49 916 Da, Table 1) and for VEGF121 (peak at 17 224 Da, Table 2).1,^12^

Immunohistologically, when analyzing all 165 glioblastoma cysts separately, in 139 (84%) the expression of basigin was more prominent in the cyst wall than in solid tumor tissue, as seen by a staining quotient of 1 or higher (Fig. 2, Table 3). This was also the case for ferritin in 107 cysts (65%), whereas less than half of the cysts (77, 47%) displayed MT in the cyst wall. When comparing the mean cyst staining quotients for patients, the values were highest for basigin, followed by ferritin and MT (Table 3).

From a statistical point of view, no correlation was found between survival time and the expression pattern of the three proteins (Table 3). In contrast to this, younger age was confirmed as a positive prognostic factor. Patient age differed significantly between short-term survivors and long-term survivors (*p * =  0·0311), as well as between short-term survivors and the rest of the cohort (*p * =  0·0069). Median survival time after first surgery was 20 months, mean 23·9 months. Survival ranged from 2 to 102 months, the 95% confidence interval went from 13·2 to 26·8 months. Eleven patients (52·4%) survived for more than 18 months (mean age 42·8 years) and 6 patients (28·6%) for more than 24 months (mean age 41·8 years).

## Discussion

In the present study, immunohistologic staining confirmed the plausibility of the SELDI-TOF analytic algorithm and revealed new details on glioblastoma protein expression. Nevertheless, for unequivocal identification of the proteins in cyst fluid, protein sequencing, fingerprint electrophoresis, or an ELISA-based protein pull-down assay of the fluid would be necessary.

The cyst wall staining quotients also support the results of the SELDI-TOF analysis. Metallothionein, which is known to be present in glioma cells, has a molecular weight near the protein peak at 6424 but lies outside the 0·3% accuracy margin. This ‘negative’ result from SELDI-TOF analysis is consistent with a less prominent immunohistologic staining signal and a cyst to tumor ratio around 1 in most patients (Table 3). Contrary to this, the signals for basigin and ferritin are predominant in the cyst wall.

The results of the analytic approach are further supported by recent publications. CaMKII (8658 Da) and haptoglobin (43 349 Da), both predicted by the SELDI-TOF analysis (Table 2), have been shown to play roles in glioblastoma pathogenesis.13,^14^

Regarding hypotheses on glioblastoma cyst formation, SELDI-TOF proves that cyst fluid is more than merely trapped CSF.^6^ This is shown by the occurrence of protein peaks with statistically significant differences between CSF and cyst fluid. Interestingly, there is also an absence of certain protein peaks in the glioblastoma cyst fluids when compared to CSF (Table 1). This could signify complete synthesis of the cyst fluid by the tumor cells, without even partly recruiting CSF, in this way accumulating actively secreted proteins or necrotic degradation products.

When assuming a mixture of CSF and secreted fluid in the cyst, the missing CSF proteins could be tumor suppressors that have been down-regulated during malignant glioma progression.^15^ For identification of these proteins, comparison of glioblastoma cyst fluid with spinal CSF from the same patient will be needed.

The immunohistologic detection of a strong expression of basigin and ferritin in the cyst wall, together with a weak staining for MT in the same tissue specimen, supports the hypothesis of active synthesis and secretion of proteins by tumor cells (Fig. 2). It remains unsolved whether the proteins are synthesized by cells residing in the cyst wall or whether the proteins diffuse into the cells from the cyst lumen by pinocytosis or are driven by a pressure gradient.

Basigin is a 40 201 Da membrane protein with isoelectric point at 5·34, which is coded on chromosome 19p13·3. It constitutes the CD147 immunoglobin for the Ok blood group, plays a role in neural network formation, and is expressed in vascular endothelium of healthy brain. Interestingly, it has been found in the cells of glioma and medulloblastoma, but not in the proliferating vessels of malignant glioma.16,^17^ Its properties as extracellular matrix metalloproteinase inducer (EMMPRIN) can facilitate metastatic spread of tumor cells and increase resistance against chemotherapy.9,^18^ Therapeutic experiments with antisense RNA and antibodies have shown first promising results.19,^20^ Since the peak was detected when comparing cyst fluids from recurrent glioblastoma with fluids from glioblastoma, there seems to be a connection between basigin and tumor cells escaping the treatment.

The light chain of ferritin is a cytosolic 19 889 Da protein with isoelectric point at 5·52 and encoded on chromosome 19q13·33. Together with ferritin heavy chains, a 24-oligomer is formed that stores iron and makes it available for metabolism. Ferritin has been described to play a role in cellular differentiation, and experiments with rat glioma cells have found a regulatory effect of insulin on its transcription.^21^ Ferritin levels can be elevated in CSF and in serum of glioblastoma patients, and the protein was detected immunohistochemically in glioblastoma tissue.10,^22^

The molecular weights obtained by the SELDI-TOF analysis disclose the presence of the ferritin light chain in the cyst fluids, a similar peak including the ferritin heavy chain (21 226 Da) is absent. This suggests an altered subunit composition of the 24-oligomer or an aberrant production of ferritin light chains by glioblastoma cells. Employing selective monoclonal antibodies against the light chain or the heavy chain for immunohistologic staining, or a SELDI-TOF analysis of microdissected ferritin-positive tumor cells can help find the answer to this question.^8^

Metallothioneins are a group of cytosolic, cysteine-rich proteins with molecular weights around 6150 Da encoded on chromosome 16q13, which bind heavy metal ions, and have been described in glioma tissue.^11^ A regulation of the transcription of the related genes can be exerted by heavy metal ions or glucocorticoids. Most recently, a correlation between expression of MT 1-E with migration and invasion of glioma cell lines has been observed.^23^ In the present study, immunohistological staining disclosed the presence of MT-1 and -2 in glioblastoma cells, which is concordant with reports on increased occurrence of MTs in higher astrocytoma grades.^24^

The alteration of CSF proteins in tumor patients and the presence of growth promoting factors in tumor cyst fluids have been described in a number of publications.1,7,^25^ With the SELDI-TOF analysis it becomes possible to describe the exact molecular weight of these proteins and to search for their presence in tumor tissue by immunohistological staining.

## Conclusion

The protein content of glioblastoma cyst fluid is significantly different from that of non-tumor control CSF. The presence of basigin and ferritin in the cyst wall supports the hypothesis of synthesis and secretion of cyst proteins by malignant cells. The SELDI-TOF approach enables further description and identification of these proteins, heading towards definition of glioma biomarkers.

## Disclosure statement

We declare that we have not received any financial support in conjunction with the above submission to ‘Neurological Research’. We have no conflict of interest with this submission concerning relationships, affiliations, or funding.
